# Moxibustion therapy for treating patients with primary osteoporosis: A systematic review and meta-analysis protocol

**DOI:** 10.1097/MD.0000000000018226

**Published:** 2019-12-27

**Authors:** Xin Hui, Hao Wang, Qin Yao, Baixiao Zhao, Lue Ha

**Affiliations:** Beijing University of Chinese Medicine, Beijing, China.

**Keywords:** complementary medicine, moxibustion therapy, primary osteoporosis, protocol, systematic review

## Abstract

**Background:**

Primary osteoporosis (POP) is a common disease among elderly, which increase the risk of fracture and impact to the quality of life. As a Chinese traditional therapy, moxibustion has been commonly applied in treating chronic musculoskeletal diseases in China. Many trails have shown that moxibustion therapy is effective in treating primary osteoporosis. The protocol aims to present the methods used to access the effectiveness and safety of moxibustion therapy for patients with primary osteoporosis.

**Methods:**

The following databases will be searched from their inception: the Cochrane Central Register of Controlled Trails(CENTRAL), Pubmed, EMBASE, China National Knowledge Infrastructure(CNKI), Chinese Biomedical Literature Database(CBM), Chinese Scientific Journal Database (VIP database), and Wan-Fang Database. Clinical randomized controlled trials related to moxibustion therapy for treating primary osteoporosis will be included, regardless of publication status and languages. Study selection, data collection, and quality assessment will be independently conducted by 2 researchers. We will select the fixed-effects or random-effects model according to the heterogeneity assessment for data synthesis. Bone mineral density(BMD) will be the primary outcomes. Visual analogue scale(VAS), response rate, TCM Syndrome scale(TCMSS), bone gla protein(BGP), alkaline phosphatase(BALP), blood calcium(Ca), blood phosphate(P), quality of life(QOL) will be the second outcomes. If it is appropriate for meta-analysis, RevMan V.5.3 statistical software will be used. Otherwise, a systematic narrative synthesis will be conducted. The results will be presented as risk ratio (RR) with 95% confidence intervals (CIs) for dichotomous data and weight mean difference(WMD) or standard mean difference (SMD) 95% CIs for continuous data.

**Trial registration number:**

PROSPERO CRD42019129507

## 1 Introduction

### 1.1 Description of the condition

Primary osteoporosis (POP) is the most common disease, which is a systemic osteopathy characterized by bone loss, bone tissue damage, increased bone fragility, and fracture prone.[^1^,^2^] Primary osteoporosis includes postmenopausal osteoporosis (type I), senile osteoporosis (type II), and idiopathic osteoporosis (including juvenile osteoporosis). The 1994 World Health Organization (WHO) diagnostic criteria are currently adopted in clinical for defining a bone mineral density (BMD)-based diagnosis of osteoporosis. Based on the T-scores derived from BMD measurements at the lumbar spine or proximal femur, the diagnosis is classified as normal, osteopenia, or osteoporosis.^[3]^ POP is a bone disease associated with aging. At the global level in 2019, approximately 9% of people are aged 65 or over.^[4]^ With the world population become aged, osteoporosis has been seen one of the hot interesting of whole globe public sanitation.

Osteoporotic fracture refers to the fracture that occurs immediately after minor trauma or daily activities. It is a serious consequence of osteoporosis. Osteoporotic fracture is one of the main causes of disability and death in elderly patients. POP not only leads to a significant decline in the quality of life of patients, but also requires a lot of manpower, material and financial resources for the medical treatment and nursing of osteoporosis and fracture, resulting in heavy family, and social burdens. It is reported that medical costs for primary osteoporosis in China will reach 10.1 billion US dollars in 2015 at least, and are expected to reach 93.73 billion US dollars and 115.74 billion US dollars in 2035 and 2050.^[5]^

### 1.2 Description of the intervention

Moxibustion, a common traditional Chinese therapy, has been used in China and other Asia countries for millennia.^[6]^ Moxibustion therapy generally applied for treating POP in clinical practice involve either traditional moxibustion with moxa floss (moxa stick), or indirect moxibustion, achieved by placing insulating meterials such as ginger, garlic, Chinese herbs, salt between the acupoints, and moxa cone.[^7^,^8^,^9^,^10^,^11^,^12^,^13^,^14^] The leaves of *Artemisia argyi* or mugwort called *ai-ye* in Chinese, which is the main raw material used for moxibustion.[^6^,^15^] Additionally, some Chinese herbs also can be mixed in moxa floss such as thunder-fire moxibustion.^[8]^ Traditional moxibustion therapy in clinical application has also derived many moxibustion methods suitable for different diseases, including heat-sensitive moxibustion, *Du*-moxibustion(spreading moxibustion), navel moxibustion, and other partition moxibustion. According to traditional Chinese medicine theory, moxibustion can warm the channels, activate blood circulation and promote *qi* circulation.^[6]^ Although moxibustion is widely used in China and other Asia countries, it is undeniable that there are some potential safety problems, including allergic reactions, burns, and infections.^[16]^

Many clinical studies of moxibustion for people with POP have indicated that it could increase bone mineral density (BMD), alleviate pain, and improve quality of life.[^7^,^8^,^17^,^18^,^19^,^20^,^21^,^22^] Clinical studies and experimental studies have shown that moxibustion might improve osteoporosis by regulating bone metabolism index, endocrine, and protein expression levels to achieve homeostasis of bone metabolism, enhancing osteoblast activity, promoting bone formation, and inhibiting osteoclast activity, inhibiting bone resorption.[^23^,^24^,^25^]

### 1.3 Why it is important to do the review

Moxibustion therapy stemmed from thousands of years ago and it has been extensively used in chronic musculoskeletal diseases. Dozens of studies have reported the effect of moxibustion therapy in treating primary osteoporosis.[^7^,^18^,^19^,^20^,^21^,^22^] A recently published systemtic review on moxibustion for POP found limited evidence supporting the effectiveness. In recent years, numbers of new randomized controlled trials have been published, which have not been included in the previous review. Moreover, we have adopted a more comprehensive retrieval strategy and included more comprehensive articles. We used a transparent and clearly defined systematic method to comprehensively evaluate the evidence. This systematic review will help to inform medical practitioners, researchers, and patients the safety and effectiveness of moxibustion for POP.

### 1.4 Objectives

To systematically evaluate the effectiveness and safety of moxibustion therapy for people with primary osteoporosis.

## 2 Methods and analysis

This protocol has been drafted under the guidance of the Preferred Reporting Items for Systematic Reviews and Meta-analysis Protocols (PRISMA-P) and Cochrane handbook for systematic reviews of interventions.^[26]^ This systematic review will be conducted from July 1, 2019 to November 31, 2019. Until then, we will make all reviewers to receive a consistency training, so that all reviewers have a basic understanding of the background, purpose, and process of the review.

### 2.1 Inclusion certain for study selection

#### 2.1.1 Types of studies

Without restrictions on language and publication status, all the randomized controlled trials (RCTs) will be included. Randomized cross-over trials will be included only phase 1 data.

#### 2.1.2 Types of participants

Patients with primary osteoporosis that including postmenopausal osteoporosis and age-related osteoporosis, regardless of sex, race, marital status or education, and economic status. Trials will be included which applying validated diagnosed criteria, for instant, WHO/NIH/OSHK guideline, Chinese guidelines for diagnosis, and treatment of primary osteoporosis.[^2^,^3^,^27^]

#### 2.1.3 Types of interventions and comparisons

Moxibustion therapy is a kind of therapy that directly or indirectly stimulates the body surface acupoints or specific parts by igniting the moxa floss (e.g., moxa cone, moxa stick) mainly made of *Artemisia argyi* leaves. Moxibustion therapy includes stick-moxibustion, direct-moxibustion, and partition moxibustion. Moxibustion therapy combined with any complementary therapy will be excluded, for example, Chinese herb decoction, acupuncture, and other complementary therapy. The following treatment comparisons will be included in the analysis:

1. Moxibustion therapy compared with no treatment;
2. moxibustion therapy compared with placebo or sham moxibustion therapy^[28]^;
3. moxibustion therapy compared with other active therapy;
4. moxibustion therapy compared combined with active therapy compared with the same active therapy.

### 2.2 Types of outcome measures

#### 2.2.1 Primary outcomes

Bone mineral density (BMD) will be the primary outcomes. The dual energy X-ray absorptiometry (DXA) is widely accepted detection method.[^2^,^3^,^27^]

#### 2.2.2 Secondary outcomes

The secondary outcomes will be accessed in the review as follows:

1. Visual analogue scale (VAS)
2. Response rate
3. TCM Syndrome scale (TCMSS)
4. Biochemical indicators, including bone gla protein (BGP), alkaline phosphatase (BALP), blood calcium (Ca), blood phosphate (P)
5. Quality of life (QOL) as measured by a validated instrument (e.g. the 36-Item Short Form Health Survey (SF-36), the World Health Organization QoL (WHOQOL))
6. Adverse events in the treatment

### 2.3 Search methods in the study

The databases as follows will be searched by 2 independent review authors (HW and QY) from July 2019 to August 2019: the Cochrane Central Register of Controlled Trials (CENTRAL); PubMed; EMBASE; the Web of Science; Traditional Chinese Medicine databases; China National Knowledge Infrastructure (CNKI); Chinese Biomedical Literature Database (CBM); Chinese Scientific Journal Database (VIP database); and Wan-Fang Database. It will not be restricted in publication languages and status. Furthermore, 2 researchers will retrieve these databases again before publication to ensure that all documents are included. A search strategy for Medline database has been established on the guidance of the Cochrane handbook guidelines (Table 1). Similar search strategies will be conducted in all the other databases.[^26^,^29^]

**Table 1:**
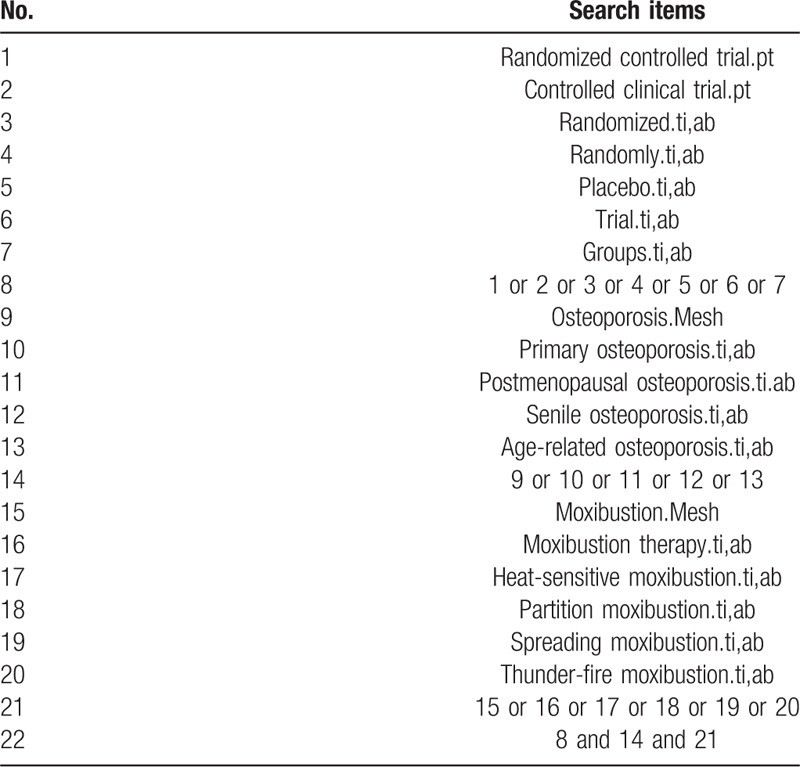
Search strategy used in PubMed.

We will search the reference lists of reviews related to moxibustion therapy and acupuncture for potential eligible studies. Besides that, we will search the conference abstracts and trial registered platforms to obtain ongoing or unpublished trials. The trial registered platforms will be searched as follows: Clinicaltrials.gov (http://www.clinicaltrials.gov) and the World Health Organization International clinical trials registry search portal (http://apps.who.int/trialsearch/).

### 2.4 Data collection and analysis

#### 2.4.1 Selection of studies

**Figure 1:**
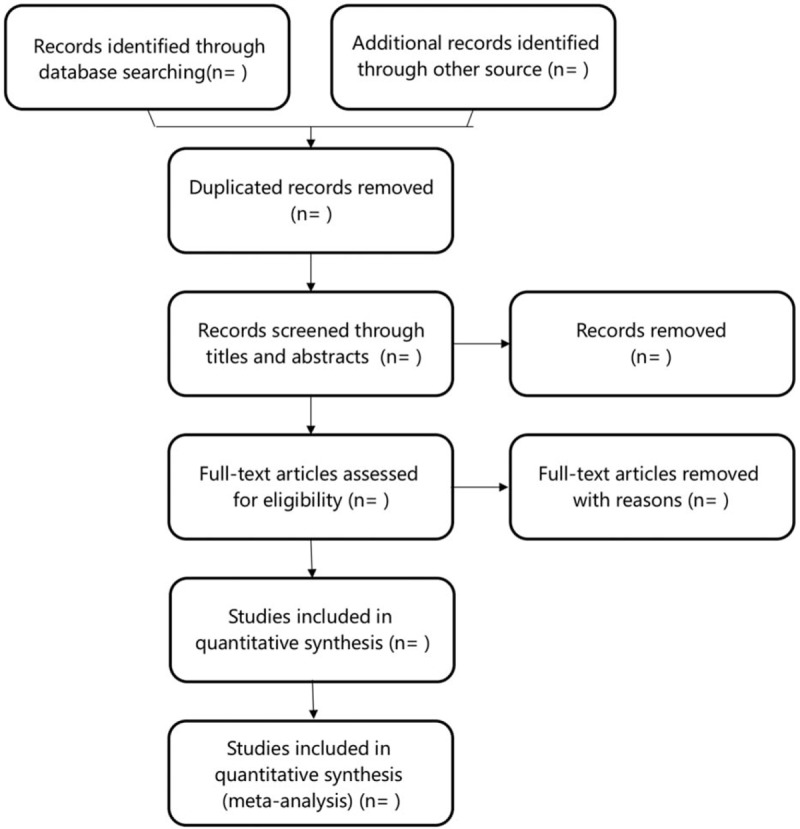
Study flow diagram.

We will enter all references into EndNote software (V.X9) to remove duplicates and manage the trials. The 2 reviewers (HW and QY) will review and screen all retrieved research titles and abstracts independently to include eligible trials. The full text will be read, if they can not identify whether the study is included or not by the above content. All the eligible articles will be labeled as “included” and “excluded”, and the reasons of exclusion will be recorded. Review authors should contact the author of the study if the article fell into the unclear category due to unclear information or missing data. Furthermore, the third review author (BXZ) will identify the study when disagreements arises. The selection process is shown in Figure 1.

#### 2.4.2 Data extraction and management

Two review authors (HW and QY) will conduct consistency training before extraction. All the authors will design an extraction form, which including general information, methods, participants, interventions, outcomes, results, adverse events and other information. In the process, any differences will be discussed by the 2 review authors (HW and QY), and if consensus is not reached, it will be decided by a third author (BXZ).

#### 2.4.3 Assessment the risk of bias in studies

The Cochrane Collaboration's tool for risk of bias assessment will be used for evaluating the methodological quality. All included studies will be assessed by 2 authors (HW and QY) independently. Disagreements will be discussed and arbitrated by the third review author (BXZ). The risk and bias assessment includes sequence generation, allocation sequence concealment, blinding of participants and personnel and outcome assessors, incomplete outcome data, selective outcome reporting, and other sources of bias. It will be classified into 3 levels: high risk, low risk, and unclear risk.

#### 2.4.4 Measures of curative effect

Weight mean difference (WMD) or standard mean difference (SMD) with 95% confidence intervals (CIs) will be applied to measure the curative effect for continuous data. Risk ratio (RR) with 95% CIs will be applied to measure the effect for dichotomous data.

#### 2.4.5 Data analysis

We will extract the data from parallel-group studies for analysis. The end of the treatment data or the end of the follow-up data will be extracted for assessment. In all studies, every single data for each outcome from every participant is collected and analysis.

#### 2.4.6 Missing data

We will contact the first corresponding authors of the included studies to get missing or insufficient trial data by telephone or email. If the data can not be obtained, we will analysis the available data and discuss the potential impact of missing data.

#### 2.4.7 Assessment of heterogeneity

Any kind of variation among studies in systematic evaluation is called heterogeneity.^[26]^ If the *I* ^2^ value is less than 50%, substantial heterogeneity will not be considered in the studies. Once the *I* ^2^ value exceeds 50%, we will investigate and report the possible reasons. Furthermore, sensitive analysis or subgroup analysis will be conducted.

### 2.5 Assessment of reporting biases

We will adopt funnel plots for assessing the reporting biases and small-study effects. If 10 or more trials studies are included in the study, a test for funnel plot asymmetry using Egger method will be conducted.^[26]^ All eligible trials will be included for funnel plots, regardless of their methodological quality.

### 2.6 Data synthesis

RevMan V.5.3 statistical software will be applied for data synthesis when a meta-analysis is allowed. We will use the RR with 95% confidence interval (CI) for dichotomous outcomes. And we will use the mean difference between treatment arms (with 95% CI) for continuous data.^[26]^ If no significant heterogeneity exists, the fixed-effects model will be used for data synthesis; otherwise, the random-effects model will be conducted for data synthesis. If quantitative synthesis is not appropriate such as insufficient RCTs or unidentified significant heterogeneity, we will conduct subgroup analysis or provide a systematic narrative synthesis to describe the characteristics and findings of the included trials.

### 2.7 Subgroup analysis

We do not have pre-subgroup plan in this review. If the studies are adequate, subgroups of different types of primary osteoporosis treated by moxibustion therapy will be considered. Furthermore, if there are significant heterogeneity exists, subgroup analysis will also be applied possibly under certain circumstances.

### 2.8 Sensitivity analysis

Where appropriate, a sensitivity analysis will be performed to evaluate the robustness of the meta-analysis results. Sensitivity analysis is used to analyze the quality of research, methodological elements, type of publication, language of publication, etc. If there is a high degree of heterogeneity, meta-analysis will be repeated to exclude low-quality or small sample studies. If sensitivity analysis changes the results, more caution must be taken in drawing conclusions. The results will be discussed according to pooled effect size.

### 2.9 Grading the quality of evidence

The Grading of Recommendations Assessment, Development and Evaluation (GRADE) will be used to evaluate evidence quality for all outcomes. The GRADE assessment classifies the quality of evidence into 4 levels: high, moderate, low, and very low.

## 3 Discussion

To date, 1 systematic review of POP has been published.^[30]^ We consider that the systematic review published for a long time, and there are more convincing RCTs, which can further evaluate the effectiveness of Moxibustion in treating POP. Our systematic review will update and provide a detailed summary of the current evidence related to the efficacy of moxibustion in treating the patients with POP. This evidence will be useful to practitioners, patients, and health policy-makers regarding the use of moxibustion in POP treatment.

## Author contributions

**Conceptualization:** Xin Hui, Hao Wang.

**Data curation:** Hao Wang, Qin Yao.

**Formal analysis:** Xin Hui, Qin Yao.

**Methodology:** Xin Hui, Hao Wang, Qin Yao, Baixiao Zhao.

**Project administration:** Xin Hui, Qin Yao, Baixiao Zhao.

**Supervision:** Baixiao Zhao.

**Validation:** Hao Wang, Qin Yao, Lue Ha.

**Writing – original draft:** Xin Hui, Hao Wang.

**Writing – review & editing:** Xin Hui, Hao Wang, Qin Yao, Baixiao Zhao, Lue Ha.
